# Important Role of Renin–Angiotensin System and Vasopressin System in Cardiovascular, Metabolic and Psychogenic Disorders in Pregnancy and Early Postnatal Life: A Narrative Review

**DOI:** 10.3390/ijms27167309

**Published:** 2026-08-16

**Authors:** Ewa Szczepańska-Sadowska

**Affiliations:** Laboratory of Centre for Preclinical Research, Department of Experimental and Clinical Physiology, Medical University of Warsaw, 02-097 Warsaw, Poland; eszczepanska@wum.edu.pl

**Keywords:** angiotensin, angiotensinogen, AT1-AA autoantibody, depression, diabetes, eclampsia, hypertension, hypoxia, pregnancy, stress, sex, vasopressin

## Abstract

The renin–angiotensin system (RAS) and vasopressin system (VAS) play an essential role in the regulation of multiple vital body functions. A survey of the literature provides evidence that the central and systemic RAS and VAS are activated during pregnancy and cooperate in the regulation of blood circulation and in the exchange of substrates and metabolites between the mother and fetus. The present review is focused on the engagement of the RAS and VAS in the regulation of blood circulation in healthy pregnant women and fetuses and in gestations complicated by cardiovascular, metabolic and psychogenic diseases. The presence of several components of the RAS and VAS, such as renin, angiotensin-converting enzymes (ACE1, ACE2), angiotensins [Ang II, Ang-(1–7)], angiotensin receptors (AT1R, AT2R), and AVP receptors (V1aR), in the placenta and the myometrium allows us to assume that the RAS and VAS play a prominent role in function of the maternal–placental–fetal unit. Gestation is also associated with the activation of components of the RAS and VAS in appropriate regions of the central nervous system that are involved in the regulation of blood pressure. Experimental studies suggest that the RAS and VAS participate in the autonomic control of blood circulation and in the regulation of emotional aspects of pregnancy and maternity. Abnormal functioning of the RAS and/or VAS was found in eclampsia, metabolic diseases (diabetes mellitus) and psychogenic disorders (anxiety, stress, and depression). Some of these abnormalities may have an immunological or genetic background. In eclampsia, the presence of specific agonistic angiotensin receptor autoantibodies (AT1-AA) and altered genotypes of AGT, ACE2, AT1R, and AT2R were reported. Polymorphism of the AVP promoter was also described. Inappropriate pharmacotherapy or toxic environmental pollution may disturb the regulation of the cardiovascular system of the mother and fetus by the RAS and VAS during gestation. Further studies are necessary to better recognize the mechanisms of action of the RAS and VAS in healthy and pathological pregnancies.

## 1. Introduction

It is generally known that multiple components of the renin–angiotensin system (RAS) and of the vasopressin system (VAS) play a fundamental role in the regulation of water–electrolyte balance and blood pressure. The vital role of the RAS and VAS becomes especially evident when the cardiovascular system is exposed to additional challenges. Pregnancy creates physiological conditions affecting functions of the cardiovascular system and of several neuroendocrine pathways, including the RAS and VAS. Cardiovascular disease of the mother or fetus usually causes ischemia, hypoxia and metabolic dysfunctions, which may further provoke the activation of the RAS and VAS [1,2,3,4,5,6]. Components of the RAS and VAS have been detected in the maternal-placenta unit, where their expression is altered in the gestational pathology [7,8,9,10]. Heart failure, hypertension, preeclampsia, metabolic disorders, obesity and diabetes mellitus may result in maternal placental syndrome (MPS) and other complications that exert harmful effects on the fetus [4,5,6,11,12,13,14]. In addition, some pregnant women may also suffer from prenatal psychological stress, which exerts a negative influence on the course of the pregnancy. Several studies emphasize the unfavorable role of psychogenic stress in the regulation of the cardiovascular system [15,16,17,18,19,20,21,22,23]. In our laboratory, we have shown that the RAS and VAS play a significant role in the modulation of cardiovascular responses to stress. We have also found that the stress-modulating effects may be significantly potentiated in cardiovascular disorders [19,20,21,22,24,25,26,27,28]. There is evidence that the regulation of the cardiovascular system by the RAS and VAS is altered during pregnancy. Thus far, the functional significance of these changes has not been comprehensively addressed. The purpose of the present review is to focus attention on the role of the RAS and VAS in the pregnant woman and fetus during prenatal and early postnatal life. Special attention is given to the role of the RAS and VAS in pregnancies complicated by cardiovascular, metabolic and psychogenic diseases. It has been shown in several clinical and experimental studies that these diseases frequently complicate the progress of the pregnancy. In addition, it has been reported that the action of specific components of the RAS and VAS is altered in pregnant women who are overweight or suffering from diabetes mellitus and/or psychic disorders [29,30,31,32,33].

## 2. Methodology

The literature search was performed with application of PubMed, EMBASE and Scopus systems, comprising articles published between 1960 and 2026. The following keywords or their combinations were considered for detailed searching: aldosterone, angiotensin, anxiety, copeptin, depression, diabetes, eclampsia, fetus, glucocorticoids, heart failure, hypertension, hypoxia, insulin, obesity, preeclampsia, pregnancy, prorenin, prorenin receptor, reactive oxygen species, renin, stress, sympathetic system, and vasopressin. Eligibility of the studies discussed in the review was evaluated according to: (1) significance of the study for comprehension of physiological role of the renin–angiotensin system and vasopressin system in the regulation of the cardiovascular functions during pregnancy and early postnatal life, (2) importance of the study for better comprehension of the role of RAS and VAS in generation of cardiovascular pathophysiology during pregnancy, and (3) reliability of methods applied in the study.

## 3. Overview of Cardiovascular Regulation in Pregnancy

After fertilization, the embryo implants in the superficial layer of the endometrium. The onset of implantation initiates the formation of the trophoblast, which differentiates to the external cell layer known as the syncytiotrophoblast and to the inner layer forming the cytotrophoblast [34]. Pregnancy requires the adjustment of functions of the cardiovascular, respiratory and excretory systems of the mother to the increasing demands of the developing fetus for nutrients and oxygen and for the removal of metabolites. Some women suffer from psychogenic stress, which imposes an additional challenge. Growing attention is being directed to the essential role of neuroendocrine factors and stress in the appropriate development of the maternal–placental–fetal unit [35]. Pregnancy is associated with altered secretion of several neuroendocrine factors, which play a crucial role in the appropriate development of the fetus [35,36,37,38,39,40,41,42,43]. Among them are components of the RAS and VAS that are known to play a significant role in the regulation of the sensitivity of the cardiovascular system to stress [19,20,21,22,23].

There is evidence that stress may exert negative effects on the development of the brain of the fetus. Experimental studies on sheep have demonstrated that the isolation stress applied in the early period of gestation influences the development of the fetal brain. It has been shown that this effect is related to the decreased synaptic density of the cortical grey matter and the hippocampus and to impaired myelination of the white matter [41]. It is also known that pregnancy is associated with increased activity of the hypothalamic–pituitary–adrenal axis (HPAA) and with enhanced secretion of glucocorticoids. It has been shown that exposure of the neonate to glucocorticoids during prenatal life may influence the sexual differentiation of the brain regions participating in the generation of the cardiovascular responses and stress–immune responses of the offspring. Strong evidence indicates that an excessive or insufficient prenatal exposure to glucocorticoids and mineralocorticoids can detrimentally affect the development of the offspring [42,43,44]. Inflammation and stress can additionally increase the secretion of glucocorticoids and cytokines [45,46]. Growing evidence shows that various components of the RAS and VAS and other neuroendocrine compounds may also play a significant role in the regulation of maternal psychogenic stress [47,48,49,50,51,52,53]. There is also evidence that preterm delivery, which causes the premature withdrawal of neuroendocrine factors such as components of the RAS and VAS, may disturb the regulation of blood circulation and development of the fetus [51,52,53].

## 4. Renin–Angiotensin System in Pregnancy

**Figure 1:**
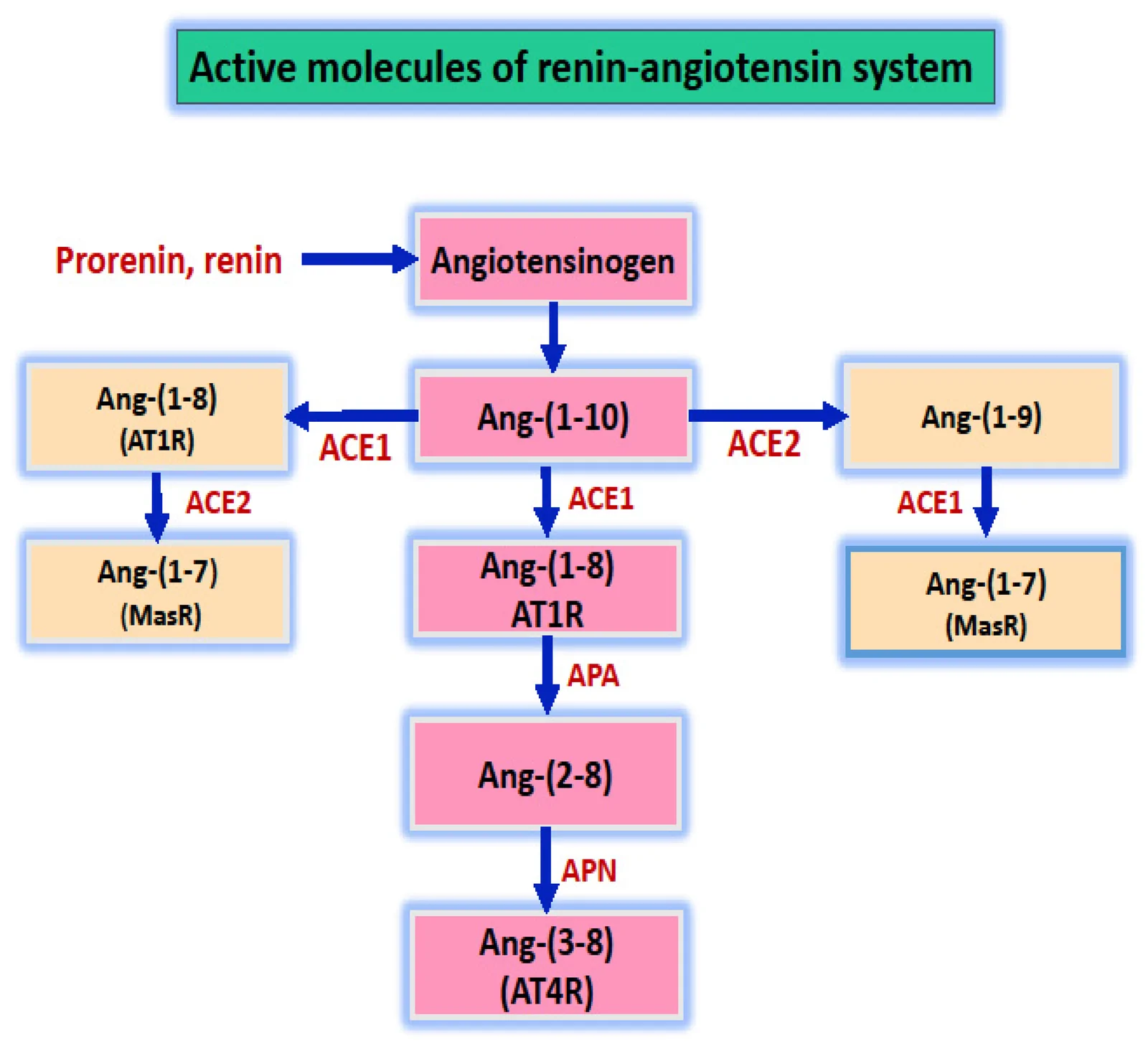
Compounds forming renin–angiotensin system (RAS) and active in pregnancy and early postnatal life. Abbreviations: ACE1—angiotensin-converting enzyme of type 1; ACE2—angiotensin-converting enzyme of type 2; Ang—angiotensin; APA—aminopeptidase A; APN—aminopeptidase N; AT1R—angiotensin receptor of type 1; AT4R—angiotensin receptor of type 4; MasR—Ang-(1–7) receptor.

The renin–angiotensin system (RAS) is a rich complex of enzymes, substrates and receptors present in several organs [54,55]. It includes preprorenin, prorenin, renin, angiotensinogen (AGT), angiotensin 1–10 (Ang I), angiotensin 1–8 (Ang II), angiotensin 2–8 (Ang III), angiotensin Ang-(1–9), angiotensin 1–7 [Ang-(1–7)], and angiotensin 3–7 [(Ang IV, Ang-(3–7)]. As shown in Figure 1, prorenin is converted to renin, which acts on its substrate AGT and cleaves Ang-(1–10). Ang-(1–10) can be converted either to Ang-(1–9) by the action of angiotensin-converting enzyme 2 (ACE2) or to Ang-(1–8) by the action of angiotensin-converting enzyme 1 (ACE1). Ang-(1–7) can be produced either from Ang-(1–9) through the action of ACE1 or from Ang II by the action of ACE2. Ang-(1–8) can also be converted to Ang-(2–8) (Ang III) by aminopeptidase A (APA). Ang-(2–8) can be converted to Ang IV by aminopeptidase N (APN). Angiotensin peptides act on AT1 receptors (AT1Rs), AT2 receptors (AT2Rs), Mas receptors (MasRs), and AT4 receptors (AT4Rs). AT4R is also known as insulin-regulated membrane aminopeptidase (IRAP) (Figure 1).

Multiple studies provide evidence that pregnancy significantly influences the function of the renin–angiotensin system. Ang II and Ang-(1–7) are present in the uterus, and their expression varies during gestation [7,56,57,58,59,60,61,62,63]. Studies on nulliparous, pregnant, and postpartum sheep provided evidence that the myometrium of nulliparous sheep expresses mainly AT2R. During pregnancy, AT1R dominates; however, five days after parturition, AT2R dominates again [58]. Experiments on Sprague Dawley rats have demonstrated the presence of the following genes of the RAS in the placenta: angiotensin-converting enzyme (*Ace*), angiotensin-converting enzyme 2 (*Ace2*) and angiotensin II receptor 1A (*AT1a*). The expression of these genes is upregulated at day 20 of gestation [59]. Studies on human embryos and fetal samples revealed the expression of mRNAs of angiotensinogen, renin, ACE, AT1R and AT2R in the kidney, the adrenal gland, the heart, and the liver [60,61]. It has been shown that during pregnancy, the expression of specific components of the RAS is influenced by environmental factors. For instance, significant elevation of ACE2 expression was found in the uterus, the placenta and the kidneys of Sabra rats maintained on a high-sodium diet [62].

**Figure 2:**
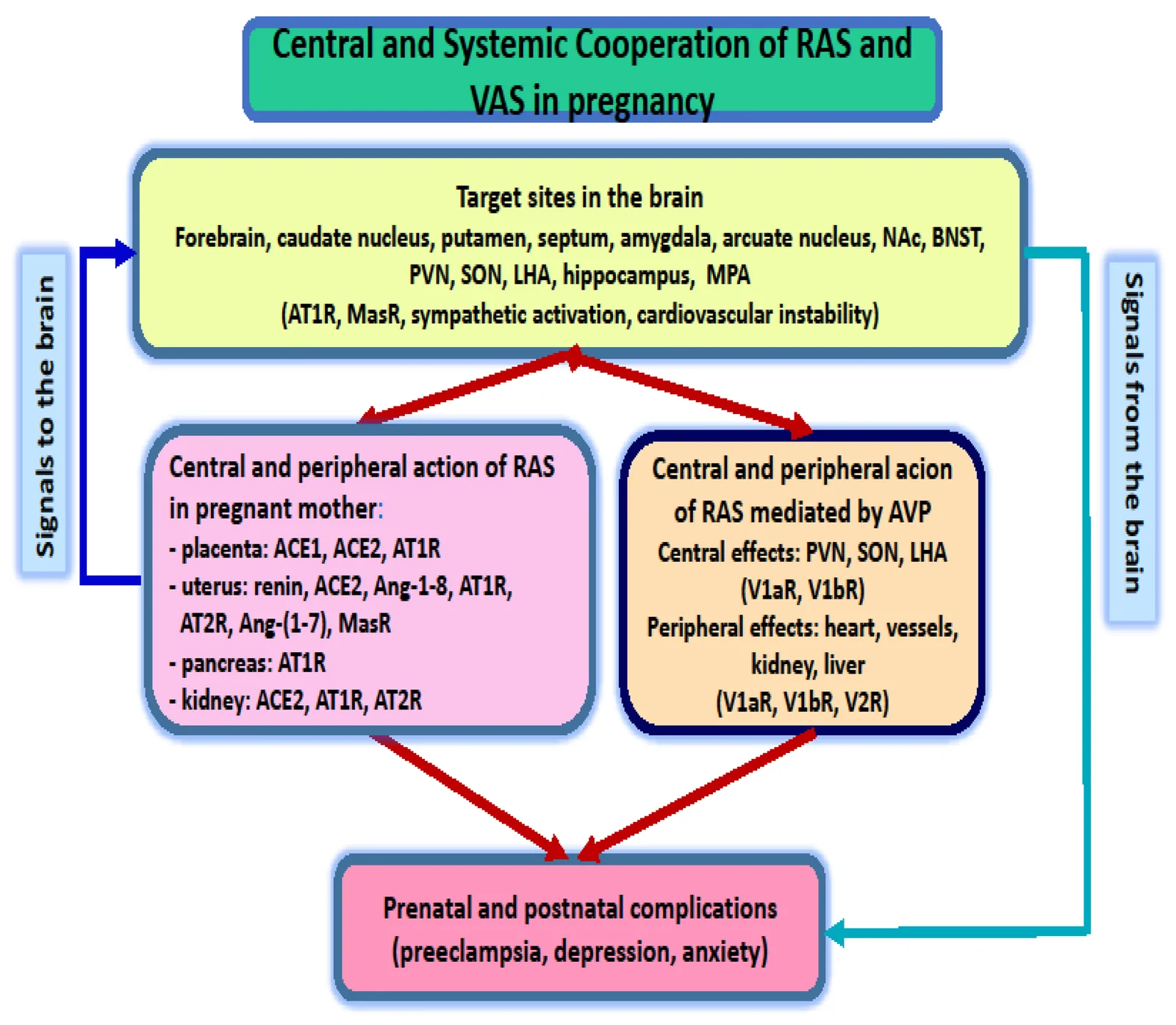
Cooperation of central and systemic components of renin–angiotensin system (RAS) and vasopressin system (VAS) in pregnancy and in early postnatal life. Abbreviations: ACE1—angiotensin-converting enzyme of type 1; ACE2—angiotensin-converting enzyme of type 2; Ang—angiotensin; AT1R—angiotensin receptor of type 1; AT2R—angiotensin receptor of type 2; BNST—bed nucleus of the stria terminalis; MasR—angiotensin-(1–7) receptor; LHA—lateral hypothalamus; MPA—median preoptic area; NAc—nucleus accumbens; PVN—paraventricular nucleus; SON—supraoptic nucleus; V1aR—vasopressin receptor of type 1a; V1bR—vasopressin receptor of type 1b; V2R—vasopressin receptor of type 2. Blue arrows indicate transmission of signals from peripheral organs to the brain. Green arrows indicate transmission of signals from the brain to the peripheral organs.

There is evidence that pregnancy influences the regulatory function of the RAS both through systemic and central effects [6,56,62,63] (Figure 2).

Evidence from experiments on Sprague Dawley rats suggests that pregnancy alters the regulation of blood pressure through the modulation of the inhibitory effect of GABA in the PVN neurons and that this action depends on the activation of AT1R [64]. Furthermore, Ang II increases sympathetic nerve activity and heart rate in Sprague Dawley rats through an interaction with AT1aR in the brain arcuate nucleus and through the stimulation of AVP secretion. Both these effects are significantly enhanced in pregnant females [65].

Notably, long-lasting stimulation of the RAS by environmental factors may potentiate its role in the regulation of the cardiovascular system during pregnancy. For instance, a high-salt diet was found to elicit remarkable changes in the structure of cardiac myofibrils and mitochondria. It also alters the expression of AT1R mRNA, AT1Ra protein and AT1Rb protein in fetal Sprague Dawley rats [66]. In addition, prolonged elevation of blood Ang II levels in pregnant rats receiving high-salt diet results in the elevation of blood pressure and in the reduction of the relative weight of the left cardiac ventricle in the offspring [67]. Recently, it has been shown that unpredictable prenatal stress elicits significant elevations of AT1R and NADPH oxidase complexes (NOX2 and NOX4) in the liver of pregnant Wistar offspring [68].

It is likely that the RAS plays an essential role in the regulation of the cardiovascular system of the fetus during hypoxia. It has been shown that hypoxia elicits in the fetus the release of renin, angiotensin and aldosterone. It also increases blood pressure and bradycardia. Presumably, these effects are mediated by the stimulation of peripheral chemoreceptors, because they could be significantly reduced by sinoaortic denervation [69]. More recently, experiments on mice have shown that prenatal exposure to hypoxia elicits an increase in blood pressure, induces cardiac fibrosis, and elevates renin mRNA and AT1R mRNA expression. Interestingly, it appears that these effects are sex-dependent, as they were present in the male but not in the female offspring [70]. The expression of ACE2 mRNA in the human placenta is reduced in growth-restricted fetuses. Therefore, it has been deduced that placental ACE2 plays a role in the regulation of fetal growth [71]. In support of this assumption is the recent finding that in human placental villous explants, ACE2 effectively reduces the expression of prooxidative enzyme NADPH and increases the expression of catalase and glutathione peroxidase, which act as antioxidative compounds [72].

Indirect evidence indicates that Ang II participates in the restructuring of the uterus. Experiments performed on endometrial tissue show that Ang II promotes the proliferation of endometrial stromal cells (ESCs). It also upregulates the expression of Alpha-Smooth Muscle Actin (a-SMA), TGF-β1 and IGF-1. These processes could be effectively reduced by Ang-(1–7) [73]. There is also evidence that the effects of angiotensin peptides in pregnancy can be modulated by steroid hormones. For instance, experimental studies on ewes revealed that in utero administration of dexamethasone during early gestation increases the expression of ANG mRNA, AT1R mRNA and AT2R mRNA in the fetal kidney. Furthermore, treatment with dexamethasone potentiates the unfavorable effects of Ang II in the kidney [52].

It has been shown that alcoholism significantly challenges the health of the mother and the fetus [74]. Children born by mothers consuming excessive amounts of alcohol exhibit abnormal regulation of the heart rate by the autonomic nervous system and neuroendocrine factors [75,76,77]. It is highly possible that the brain angiotensinergic system may contribute to these dysregulations, as it has been shown that prenatal exposure to alcohol enhances the expression of AT1R mRNA and ACE mRNA in the hippocampus of C57Bl/6 mice [77].

## 5. Vasopressin System in Pregnancy

**Figure 3:**
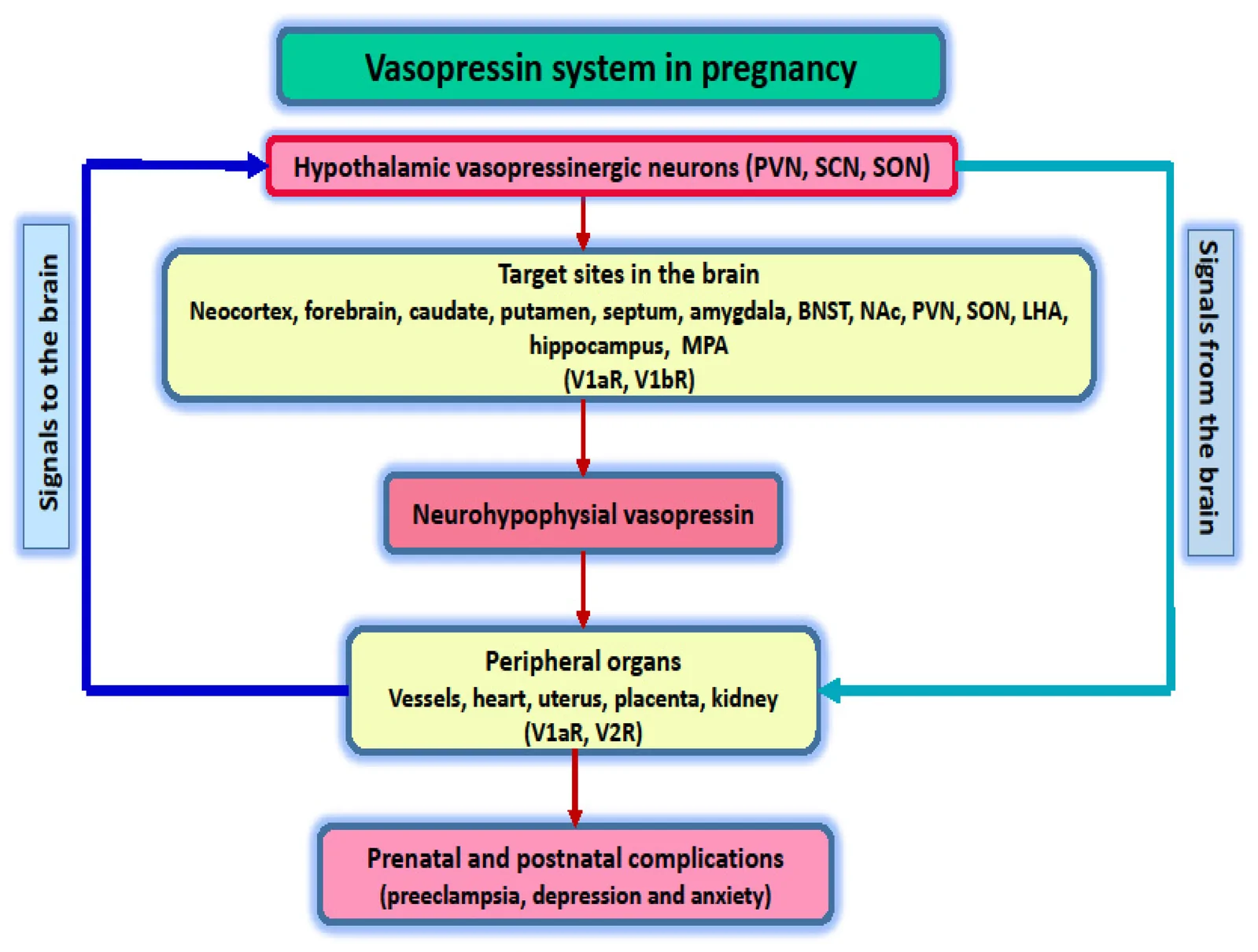
Role of vasopressin system in pregnancy and early postnatal life. Abbreviations: BNST—bed nucleus of the stria terminalis; LHA—lateral hypothalamus; MPA—median preoptic area; NAc—nucleus accumbens; PVN—paraventricular nucleus; SON—supraoptic nucleus; V1aR—vasopressin receptor of type 1a; V2R—vasopressin receptor of type 2. Blue arrows indicate transmission of signals from peripheral organs to the brain. Green arrows indicate transmission of signals from the brain to the peripheral organs.

The vasopressin system (VAS) encloses neurons of the supraoptic nucleus (SON), the paraventricular nucleus (PVN), the suprachiasmatic nucleus (SCN), and their receptors. The vasopressin gene consists of exons encoding sequences of N-terminal signal peptide, vasopressin, neurophysin II and copeptin. As vasopressin and copeptin are released in equimolar quantities, copeptin is frequently used as the biomarker of AVP [8]. Vasopressin receptors are present in several regions of the brain and in peripheral organs, mainly in the heart, vessels, lungs, kidneys, and gastrointestinal tract. The effects of AVP are mediated by V1a receptors (V1aRs), V1b receptors (V1bRs), and V2 receptors (V2Rs). The secretion of vasopressin is significantly intensified by hyperosmolarity, hypovolemia, hypotension, hypoxia and exposure to stress, while hypoosmolarity and hypervolemia exert inhibitory effects [25]. Pregnancy is associated with significant activation of the VAS, and this is related both to the increased secretion of vasopressin and to the enhanced stimulation of vasopressin receptors (Figure 3).

It has been shown that the birth process occurring via vaginal delivery causes significant elevation of AVP in the blood of the mother and infant [78,79]. The AVP concentration in the blood of the newborn is significantly higher than that in the blood of the mother; therefore, it has been concluded that the fetus is able to secrete vasopressin [78]. The presence of V1aR in the placenta has also been reported [8,10]. In addition, it has been shown that pregnancy is associated with elevated expression of specific components of the VAS in tissues located outside of the reproductive system, including the brain. For instance, in a study on pregnant rabbits, it has been shown that in late pregnancy and in the early postpartum days, the number of immunoreactive neurons expressing AVP is significantly elevated in the PVN, the SON, and the lateral hypothalamus (LHA) but not in the SCN [38].

It is likely that the secretion and action of AVP in the mother is influenced by the intensity of stress experienced during pregnancy and by the mode of delivery. For instance, measurements of AVP and copeptin levels in arterial umbilical cord blood samples collected immediately after births following vaginal delivery (VD) or elective cesarean section (ECS) revealed that VD is associated with twofold higher levels of AVP and copeptin than ECS delivery. It should be noted that vaginal delivery is more stressful. Interestingly, the mode of delivery did not influence oxytocin concentration in the umbilical blood samples of infants [79].

There is evidence that AVP may regulate the emotional aspect of maternal attitude. Experiments on rats have shown that in the peripartum period, the expression of V1aR in the brain is positively correlated with the aggressive behavior of the mothers. Especially high expression of these receptors is found in the PVN, the central amygdala and the lateral septum, i.e., in the structures playing a significant role in the regulation of emotions [80,81,82]. Studies on rats revealed that acute prenatal stress significantly increases the expression of V1aR in the lateral septum and the BNST and that it impairs social memory [82,83,84]. Similarly, studies performed on titi monkeys provided evidence that during the postnatal weeks, the expression of V1aR binding is reduced in the hippocampus, the medial preoptic area (MPA), the lateral septum and the superior colliculus. In primates, these regions of the brain are involved in the regulation of emotional behavior, attention, learning and memory. Accordingly, it has been suggested that AVP plays an essential role in the regulation of parental care [84]. Recently, it has been shown that maternal stress associated with the fear of air pollution significantly influences social behavior and causes a significant increase in *V*1*aR* mRNA in the nucleus accumbens (NAc) of female mice neonates [85]. Stress also enhances the secretion of glucocorticoids and cytokines [46,47,48,49,50,51,86,87], which are known to interfere with the action of vasopressin [24].

Prenatal exposure to alcohol and early life adversity significantly influence AVP mRNA and oxytocin mRNA expression in the hypothalamus of rats. It is possible that the stress-promoting addictions and habits of pregnant mothers, such as alcoholism or high consumption of carbohydrates and fat, may result in the formation of shorter telomeres in neonates, reducing the safeguarding of the fetal chromosomes and disturbing the genomic stability of the fetus [88].

## 6. Renin–Angiotensin System in Pathogenesis of Pregnancy

It is generally known that cardiovascular and metabolic diseases significantly worsen the prognosis of pregnancy. The most frequent complication is preeclampsia, which usually appears in the third trimester of pregnancy and is associated with hypertension and stroke and with kidney failure. Preeclampsia also causes impaired perfusion of the placenta, and this may worsen the exchange of nutrients and catabolites between the mother and the fetus.

### 6.1. Renin–Angiotensin System and Preeclampsia

Strong evidence indicates that specific components of the RAS play a significant role in the pathogenesis of preeclampsia and eclampsia. In pregnancy associated with hypertension, the expression of AT1 receptors in the endothelium of vessels in the placenta is elevated [6]. Increased production of inflammatory cytokines and ROS, as well as restricted fetal body growth, was also reported [89,90,91,92,93,94].

There is evidence that the engagement of the RAS in blood pressure regulation during eclampsia has an immunological background or specific genotype. It has been shown that some patients with preeclampsia and eclampsia develop agonistic AT1R autoantibodies (AT1-AA), which interact with AT1R and present altered genotyping of angiotensinogen and complement [95,96,97,98,99,100]. Clinical and experimental studies provide evidence that AT1R-AA exert effects similar to those observed in preeclampsia. This is especially evident during the activation of complement, production of ROS, stimulation of NADPH oxidase, and formation of nitrite [95,96,98,99]. Experiments on Sprague Dawley rats revealed that the administration of AT1-AA by osmotic minipumps to pregnant females causes a significant increase in blood pressure and elevation of blood levels of progesterone and T regulatory cells. The authors suggested that AT1-AA belong to the family of risk factors that predispose progeny to the development of hypertension and symptoms of preeclampsia [99,100]. It has been reported that women with a history of preeclampsia have elevated AT1-AA levels during the 5 years after delivery. They also manifest enhanced pressor responsiveness to Ang II and reduced responsiveness to AT2R–mediated vasodilation. These findings suggest that the prolonged postpartum action of AT1R-AA may also play a significant role in long-term blood pressure regulation [99]. Experimental studies show that the inhibition of AT1-AA exerts beneficial effects in preeclampsia. For instance, studies on Sprague Dawley rats with a preclinical model of preeclampsia reveal that the application of AT1-AA inhibitor reduces the elevation of arterial blood pressure, improves the glomerular filtration rate, and reduces oxidative stress [100]. AT2R mRNa was also identified in the cytotrophoblast [101]. Thus far, there is no direct evidence for the functional engagement of AT2R in processes occurring during eclampsia.

*Angiotensin*-(1–7). Several studies argue that there is significant engagement of Ang-(1–7) in the regulation of the cardiovascular system in pregnancy and altered functioning of the Ang-(1–7)/Mas unit in eclampsia [102,103,104,105,106,107,108,109,110,111]. It has been shown that the concentration of Ang-(1–7) in the plasma and urine of pregnant women is elevated [102,103] and that its level is reduced in preeclampsia. Immunocytochemical studies demonstrated that ACE2 and Ang-(1–7) are present in the placenta of pregnant women and that the expression of ACE2 is increased in the umbilical arterial endothelium of pregnant women with preeclampsia. Moreover, altered expression of Ang-(1–7)/Mas components has been found in specific tissues of women with preeclampsia. Namely, the presence of ACE2 and Ang-(1–7) in the syncytiotrophoblast, cytotrophoblast and endothelium of vessels of pregnant women has been reported. The expression of Mas was found to be lower in podocytes obtained from women with preeclampsia than in podocytes of normotensive pregnant women. It was also shown that the downregulation of MasR in women with preeclampsia could be reversed by co-culture of the podocytes with Ang-(1–7) [105]. Experimental studies on rats show the elevated expression of AT1R-(1–7) components in the uterus of hypertensive pregnant rats at late gestation. Accordingly, it has been suggested that this effect may be a compensatory mechanism which prevents the elevation of utero-placental vascular resistance and other negative complications of hypertension [103,104,105,106]. It has also been shown that the pharmacological blockade of Mas in Wistar rats or the genetic knockout of MasR in mice reduces the pregnancy-induced hypertrophy of cardiomyocytes. It is possible that this effect was associated with increased deposition of collagen III [107]. Furthermore, it has been found that the treatment of pregnant hypertensive rats with Ang-(1–7) during gestation reduces the increase in systolic blood pressure in adult offspring and decreases the cardiomyocyte diameter and fibrosis in the left ventricle of the offspring [108]. It has been shown that the treatment of pregnant rats with Ang-(1–7) exerts a significant effect on the cardiovascular parameters of the progeny and may probably counterbalance the negative cardiovascular and inflammatory consequences of hypertension [109]. Thus, it appears that the operation of the Ang-(1–7)/MasR axis during pregnancy may have an impact on the well-being of the progeny. It should also be noted that studies on human beings reveal that adolescents born prematurely by obese women have higher blood levels of Ang II and reduced levels of Ang-(1–7). This was associated with higher blood pressure and lower body weight [110]. Altogether, these studies indicate that pregnancy causes significant, not yet fully recognized alterations in the function of the Ang-(1–7) axis that may oppose the unfavorable effects of Ang II [111].

*Prorenin and (pro)renin receptor.* In several studies, elevated levels of circulating prorenin, the (pro)renin receptor (P)RR and the soluble prorenin receptor (sP)RR have been reported in pregnancy [112,113,114,115,116,117,118,119,120]. The prorenin receptor belongs to the family of transmembrane receptors. The (P)RR gene was detected in the placenta and in several other organs, including the kidney, pancreas, and liver. The soluble (P)RR can be released to the extracellular space and may serve as the circulating modulator of other cells [114]. Elevated levels of the (P)RR and s(P)RR have been found in pregnancies complicated by gestational diabetes mellitus and preeclampsia [116,117,119,120]. Some evidence indicates that the (P)RR may play an essential role in the regulation of the proliferation, migration and invasion of the trophoblast. It is essential to note that lowered availability of the (P)RR results in a reduced fetal–placental weight ratio; therefore, it is likely that the (P)RR plays an essential role in the exchange of substrates in the placenta [118]. However, it should also be noted that elevated circulating levels of maternal s(P)RR have been found in cases of fetal growth restriction [114]. Thus far, the role of prorenin during gestation complicated by cardiovascular and metabolic diseases is not yet sufficiently known and requires further investigations.

*Neonates.* A comparison of renin and aldosterone levels in the serum of neonates born at term and preterm did not show significant differences between these groups immediately after birth; however, on the fifth postnatal day, the levels were significantly higher in the preterm-born infants than in the infants born at term. Presumably, the elevated secretion of renin and aldosterone may account for the greater cardiovascular instability of the preterm-born neonates [116]. It appears that postnatal health may depend on the genotype of RAS components. For instance, it has been shown that the presence of the ACE2 gene rs2074192 T allele in the neonate is associated with the smaller growth of the baby [121].

### 6.2. Renin–Angiotensin System in Metabolic Disorders

There is evidence that altered stimulation of angiotensin receptors contributes to inappropriate regulation of metabolism and blood pressure during pregnancy complicated by obesity and/or diabetes mellitus. An elevated level of circulating autoantibodies (AT1-AA) was found in pregnant women suffering from type 1 diabetes, type 2 diabetes, and gestational diabetes mellitus [122,123]. Experiments on pregnant mice with streptozotocin-induced diabetes mellitus (C57BL/6) revealed that the diabetic mice exhibited enhanced sympathetic nerve activity, elevated plasma norepinephrine levels and higher expression of AT1 receptors in the PVN. These alterations are associated with decreased methylation of the promoter region of AT1R. It is likely that the crucial role of Ang II in inappropriate regulation of the cardiovascular system in metabolic diseases results from generation of oxidative stress. In support of this assumption is the finding that bilateral PVN infusion with AT1R antagonist losartan, or chronic application into the PVN of tempol, an antioxidant compound, effectively normalized the activity of the sympathetic system in pregnant mice [123]. It is also essential to note that reactive oxygen species are elevated in the placental tissue of patients with gestational diabetes mellitus [124]. Experiments on male Wistar rats demonstrate that active immunization with AT1-AA IgG decreases the secretion of insulin and increases apoptosis and autophagy in the insulinoma cell line (INS-1), which expresses characteristics of pancreatic β-cells [125]. An insertion/deletion (I/D) polymorphism of a 287 bp Alu repetitive sequence in the intron 16 of ACE has been identified in Asian-Indian women with gestational diabetes mellitus [126]. This suggests that the abnormal functioning of the RAS in diabetes mellitus and metabolic syndromes may have a genomic origin. As discussed above, pregnancies complicated by diabetes mellitus are associated with elevated blood concentrations of (P)RR in pregnant mothers and neonates. Elevated blood levels of Ang II and lowered concentrations of Ang-(1–7) were found in prematurely born overweight/obese adolescents, which may suggest an impact of prematurity on the programming of the RAS and body mass in adulthood [111].

## 7. Vasopressin System in Pathogenesis of Pregnancy

### 7.1. Vasopressin System and Preeclampsia

*Parturition and preeclampsia.* Almost fifty years ago, it was shown that during vaginal delivery, AVP levels are elevated in the blood of the human umbilical cord [127]. Subsequent studies demonstrated elevated levels of AVP and copeptin in preeclampsia [128,129]. Experimental studies performed on C57BL/6 mice models of eclampsia provided evidence that the continuous administration of AVP during pregnancy provokes symptoms that are characteristic of preeclampsia in offspring. It also influences the development of the neocortex, the dorsal forebrain and the caudate putamen, i.e., regions of the brain that participate in processes of attention, learning, and memory. It also intensifies anxiety-like behavior. The above effects are sex-specific and are associated with increased expression of HIF1α and SOD1 (superoxide dismutase) and with decreased expression of IGF1 [130]. AVP-dependent susceptibility to preeclampsia appears to be genetically determined. For instance, in the study by Erfanian et al. [131], polymorphism rs3729965 of the AVP promoter correlated with preeclampsia and body mass index (BMI).

*Other forms of hypertension.* Experimental studies show that the functioning of the AVP system in pregnancy may also be affected in other forms of hypertension. For instance, experiments on spontaneously hypertensive rats (SHRs) have demonstrated that in non-pregnant SHR dams, the expression of AVP, V1aR, and V1bR genes in the PVN and SON, as well as blood levels of AVP, was higher than in Wistar normotensive dams. However, pregnant SHRs exhibited a lower plasma AVP concentration and reduced AVP gene expression in the SON than the normotensive dams, whereas V1bR expression in the SON was elevated. These changes were associated with a higher variability of cardiovascular parameters. Altogether, these results suggested that pregnancy causes significant reorganization in the functioning of the VAS. Presumably, these changes resulted from the reduced secretion of AVP by the SON in pregnant SHRs and may play an essential role in cardiovascular regulation [132]. It can be hypothesized that the altered release of AVP in hypertension plays an essential role in the regulation of blood pressure by the sympathetic and parasympathetic branches of the autonomic nervous system. On the other hand, the feedback signals from baroreceptors and chemoreceptors to the SON and PVN may also be altered.

### 7.2. Vasopressin System in Metabolic Disorders

As discussed in other articles, growing evidence draws attention to the essential role of vasopressin in the pathogenesis of diabetes mellitus and in metabolic syndromes [133,134,135,136,137,138]. It appears that the inappropriate functioning of the VAS may contribute to the development of gestational diabetes mellitus, as it has been shown that pregnant mothers suffering from diabetes mellitus have reduced serum copeptin concentrations [138]. Unfortunately, this field is not yet well recognized and requires further intense research.

### 7.3. Vasopressin System in Psychogenic Disorders

*Postpartum depression.* After childbirth, some women suffer from transient major depression, which is known as gestational postpartum depression (PPD). In 1997, van Londen et al. reported that patients suffering from depression and melancholia have higher plasma AVP levels than the healthy controls [139]. In the following years, significant associations between plasma AVP and anxiety were found in pregnant women with a family history of depression and anxiety. Similar associations were found between postpartum depression and blood AVP and copeptin levels [140,141,142]. Moreover, pregnant women with symptoms of depression and anxiety manifest a higher expression of AVP and CRH genes in the placenta, and the positive correlation between the expression of these two genes was also demonstrated [142]. Recently, experiments on mice have shown that chronic deficiency of omega-3 polyunsaturated fatty acids (PUFAs) in the diet of pregnant dams and fetuses increases anxiety and depression-related responses and elevates the expression of AVP and of V1bR in the hypothalamus and the pituitary. These results suggested that the regulation of anxiety-like behavior by AVP during pregnancy may depend on the availability of PUFAs in the diet [143].

*Other psychogenic disorders.* There is evidence that the function of the VAS may also be altered in other psychic disorders. For instance, it has been shown that some pregnant women suffering from first-episode psychosis manifest elevated expression of the genes and proteins of AVP and V1aR in the placental villi and in the decidual cells [10]. Interestingly, experiments on Sprague Dawley rats revealed that acupuncture treatment, which is applied in Eastern therapy to diminish pain and anxiety, reduces AVP immunoreactivity in the PVN of rat pups and decreases anxiety behavior provoked by maternal separation during the early (14–21 days) period of postnatal life [144].

## 8. Perspective and Future Directions

Pregnancy by itself creates new demands for the circulatory, respiratory, metabolic and excretory systems of the mother. These challenges can additionally be intensified by the coincidence of cardiovascular, metabolic and neurogenic pathologies. The present review is focused on the importance of the appropriate functioning of the renin–angiotensin system and vasopressin system in the maintenance of the optimum milieu for the pregnant mother and fetus during pregnancy and early postnatal life. It is now evident that pregnancy is associated with the activation of the RAS and VAS and with the increased synthesis and action of compounds belonging to these systems. Experimental and clinical studies provide evidence that during pregnancy, the synthesis of renin, angiotensinogen, ACE1, ACE2, AT1R, AT2R, MasR, the (pro)renin receptor, AVP, V1aR and V1bR is altered in several organs playing a crucial role in the regulation of blood circulation, metabolism and psychic comfort. In particular, these changes are prominent in the brain, the kidney, the adrenal gland, the heart and the liver [6,7,8,57,58,59,60,61,62,63,64,104,105,110]. There is also strong evidence that the elevated synthesis of renin, the (pro-renin) receptor, Ang-(1–8), Ang-(1–7) and AVP has regulatory consequences and may participate in the generation of symptoms of stress, eclampsia, and psychogenic and metabolic diseases [7,8,9,10,11,12,56,63,67,68,73,78,79,80,81,91,94,95,96,97,101,102,103,104,105,106,107,108,109,110,111,112,113,114,115,116,117,118,119,120]. As discussed below, susceptibility to the inappropriate functioning of the RAS and VAS during pregnancy may have an epigenetic or genetic background.

*Negative consequences of stress.* Usually, stress activates multiple mechanisms that protect against its potential side effects; however, strong or prolonged stressing can cause distinct reprogramming of both single cells and complex regulatory systems [10,17,18,19,20,21,22,23,41,48,50,68]. It is well established that neurogenic stress plays an essential role in the activation of the RAS and VAS and that the RAS and VAS actively participate in the modulation of the intensity of responses to stress [79,81,89,94,127]. It is also known that the activation of the RAS and VAS in pregnancy occurs in connection with the stimulation of other stress-activated partners, such as glucocorticoids, aldosterone and cytokines [34,36,49,51,66,68]. It has been shown that prenatal stress may exert negative effects on the development of the fetus [41,43,44,47,48,49,50,51]. Maternal anxiety was found to exert negative effects on the development of the fetal brain, and this was associated with decreased choline and creatinine content in the brain tissue [145,146]. There is evidence that excessive stimulation of the renin–angiotensin–aldosterone system causes damage of the endothelium and the remodeling of vessels in several organs, including the brain [145,146]. Thus, it is likely that injury of the endothelium and the inappropriate exchange of substrates and metabolites between the blood and the brain may account for the negative effects of excessive stimulation of the RAS on the development of the fetal brain. Experiments on Sprague Dawley rats have shown that chronic exposure of dams to postnatal stress significantly reduces AVP expression in the PVN and potently decreases maternal care. This suggests the important role of the VAS in the regulation of maternal behavior during stress [79,80].

There is evidence that a stress-promoting style of life markedly influences the well-being of the mother and the development of the child. For instance, for pregnant women, listening to music was found to exert positive effects on maternal stress resilience [147]. Conversely, chronic stress, such as the risk of war or epidemic infections, significantly intensified stress sensitivity, anxiety and depression in pregnant women and enhanced the fear of delivering the child [148,149,150,151,152,153,154].

*Epigenetic and genetic modifications*. It is becoming evident that sensitivity to angiotensin peptides and vasopressin may be affected by epigenetic modifications. In vitro experiments on carotid bodies of Sprague Dawley dams showed that the application of nicotine upregulates the methylation of AGT receptors [155]. Epigenetic modification may also contribute to the inappropriate functioning of the RAS during pregnancy. As discussed above, the significant role in the regulation of the cardiovascular system in preeclampsia has been attributed to AT1R autoantibodies [156]; however, it cannot be excluded that under some specific pathological conditions, the mother or fetus may produce other types of autoantibodies, which may interact epigenetically with other components of the RAS or VAS. Presumably, alcoholism, which is sometimes a significant problem during gestation [74,75,76], can be included among the group of epigenetic modifiers that effectively influences the functioning of the RAS and VAS of the mother and fetus. It is likely that several factors, such as oxidative stress, steroid hormones, immunological factors, and specific pharmacological medication, may additionally cause epigenetic modifications to the functioning of specific components of the RAS and VAS in the pregnant mother and fetus and that they may influence the teratogenic/fetotoxic profile of gestation. Thus far, this field is not yet well explored and requires further investigation.

Some studies argue that the inappropriate functioning of specific components of the RAS during pregnancy has a genetic background in certain clinical cases [97,121,157,158,159,160,161,162,163,164]. The insertion/deletion polymorphism of angiotensinogen, ACE, ACE2, and AT1R genes was described in women suffering from eclampsia [99,120,164]. Polymorphism of the RAN gene (representing the large RAS family) is not associated with recurrent pregnancy loss [161]. A correlation between polymorphism of the AVP promoter gene and preeclampsia has also been reported [129].

*Inappropriate energy turnover*. Overweight and obesity are among the frequent complications of pregnancy that can negatively influence the health state of the pregnant woman, especially when they are associated with metabolic diseases, such as diabetes mellitus. There are reasons to assume that the action of the RAS and VAS is altered in pregnancies complicated by metabolic diseases. Thus, the elevated level of AT1-AA in the blood of pregnant women suffering from diabetes mellitus strongly suggests that the epigenetic control of AT1R expression—and, consequently, sensitivity to Ang II—may be altered in diabetes mellitus [122]. Similarly, the elevated concentration of copeptin in the blood of pregnant women suffering from gestational diabetes mellitus provides evidence for the altered regulation of AVP secretion in this disease [138].

*Importance of central components of the RAS and VAS in pregnancies complicated by neurological disorders*. The presence of all components of the RAS and VAS in several brain regions and the involvement of these systems in the regulation of brain functions [23,24,27,55] strongly suggest that the improper action of central components of the RAS and VAS may account for the inappropriate regulation of blood pressure in pregnant patients suffering from neurologic diseases. Strong links, both at the genomic and epigenetic levels, in the anomalous functioning of the RAS and VAS have been described in eclampsia and preeclampsia. Anomalous functioning of the brain renin–angiotensin system has been found in Parkinson’s disease, Alzheimer’s disease, brain trauma and cerebrovascular diseases [165,166,167,168,169,170]. It has been shown that the pathogenesis of Parkinson’s disease, which causes the degeneration of dopaminergic neurons, is associated with the upregulation of the pro-oxidative/pro-inflammatory AT1 receptor axis and that excessive stimulation of AT1R may accelerate neuronal death [166]. Enhanced activation of the RAS has also been found in traumatic brain injury and in stroke [165,170].

*Unanswered questions.* In spite of significant progress in noninvasive methodology, evaluation of the role of the RAS and VAS in pregnancy and postnatal life still has several shortcomings. The deficiency of satisfactory noninvasive methods for human perinatology means that the current knowledge is largely based on experimental studies, performed on various species, and in different experimental conditions. The problem that requires extensive investigation is the role of the RAS and VAS in pregnancies associated with hypoxia of the mother or fetus, as it is known that hypoxia is an effective stimulator of the release of AVP and renin [171,172]. Presumably, interspecies differences and the diversity of experimental conditions account for the diversity of action of the various compounds that interfere with the VAS and RAS in various organs of the pregnant mother and fetus during normal and pathological pregnancies. Significant interspecies differences in reproductive physiology do not permit the transference of all results obtained in animal species to human beings. Therefore, the information is frequently fragmentary, randomly collected, and based on observations and mechanistic insight. Nevertheless, in spite of these shortcomings, the available data are sufficiently interesting to give grounds for further investigations.

During pregnancy, components of the RAS and VAS participate in several ways in the regulation of blood circulation, water–electrolyte balance, energy balance and emotional comfort. Experimental studies suggest that female and male neonates respond differently to prenatal stimulation of the RAS and VAS. Hypertension, cardiac failure, obesity, emotional instability and the treatment of these diseases during pregnancy create serious problems. The grounds for the application of ACE inhibitors, angiotensin receptor antagonists or AVP antagonists in the treatment of these disorders during pregnancy are not fully known. Undoubtedly, our knowledge of the teratogenic and fetotoxic consequences of the administration of various types of pharmacological treatments that have to be used when multiple pathologies coexist with gestation requires further intense investigation.

As noted in the present review, the RAS and VAS cooperate closely with aldosterone [146,160,164]; however, a detailed discussion of the role of aldosterone in pregnancy exceeds the scope of the present article, and the reader is advised to look for literature in other recent review articles [146,164,173,174,175,176,177,178,179,180].

## 9. Conclusions

The components of the renin–angiotensin system and vasopressin system play a pivotal role in the regulation of water–electrolyte balance, blood pressure, metabolism, and psychogenic comfort. Pregnancy requires the adjustment of the functioning of the RAS and VAS to new challenges. Multiple studies performed on human beings and animal species reveal that during pregnancy and early postnatal life, the RAS and VAS are activated in a coordinated manner and have significant regulatory potential. During pregnancy, the stimulation of the RAS and VAS is significantly affected by stress. Functions of the RAS and VAS are significantly disturbed in pregnancies complicated by cardiovascular, metabolic and neurogenic diseases, such as eclampsia, diabetes mellitus, depression and other neurologic pathologies. There is evidence that in pregnancy, several dysfunctions of the RAS and VAS may result from epigenetic or genetic abnormalities. Current evidence strongly suggests that during pregnancy, specific pharmacological therapies or environmental challenges may effectively interfere with the functions of both systems. Further studies assessing the function of the RAS and VAS in gestations complicated by cardiovascular, neurologic and metabolic diseases would be of great importance for better understanding the mechanisms of action of specific components of the RAS and VAS in the mother and fetus and for the elaboration of the safest and most effective therapeutic strategies in individual pregnancies.

## Data Availability

No new data were created or analyzed in this study. Data sharing is not applicable to this article.

## Abbreviations

ACE—angiotensin-converting enzyme of type 1, ACE2—angiotensin-converting enzyme of type 2, AGT—angiotensinogen, Ang—angiotensin, AP—area postrema, αSMA—Alpha-Smooth Muscle Actin, AT1-AA—autoantibodies, AT1R (AgtR1)—angiotensin receptor of type, AT2R—angiotensin receptor of type 2, AVP—arginine vasopressin, AVPR1a—AVP receptor of type 1a, AVPR1b—AVP receptor of type 1b, BMI—body mass index, CRF—corticotropin-releasing factor, CRH—corticotropin-releasing hormone, ECS—elective cesarean section, GABA—gamma aminobutyric acid, HIF—hypoxia-inducible factor, HPAA—hypothalamic–pituitary–adrenal axis, IGF-1—insulin-like growth factor, IL—interleukin, INS—insulinoma, IRAP—insulin-regulated aminopeptidase, IRS—insulin receptor substrate, ISI—insulin sensitivity index, LHA—lateral hypothalamus, MAP—mean arterial blood pressure, MasR—Mas receptor for angiotensin-(1–7), MPA—median preoptic area, MPS—maternal placental syndrome, NAc—nucleus accumbens, NADPH—dinicotinamide adenine dinucleotide phosphate, NOX—NADPH—nitric oxidase complex, OTR—oxytocin receptor, PPD—postpartum depression, PUFAs—polyunsaturated fatty acids, PVN—paraventricular nucleus, RAS—renin–angiotensin system, RAAS—renin–angiotensin–aldosterone system, ROS—reactive oxygen species, SCN—suprachiasmatic nucleus, SD—Sprague Dawley rat, SHR—spontaneously hypertensive rat, SOD—superoxide dismutase, SON—supraoptic nucleus, TGF-β1—transforming growth factor of type β1, TNF-α—tumor necrosis factor alpha, VAS—vasopressin system, VD—vaginal delivery, V1aR—vasopressin receptor of type V1a, V1bR—vasopressin receptor of type V1b, V2R—vasopressin receptor of type 2.
